# Administration of GnRH at Onset of Estrus, Determined by Automatic Activity Monitoring, to Improve Dairy Cow Fertility during the Summer and Autumn

**DOI:** 10.3390/ani11082194

**Published:** 2021-07-23

**Authors:** Zvi Roth, Yaron Z. Kressel, Yaniv Lavon, Dorit Kalo, David Wolfenson

**Affiliations:** 1 Department of Animal Sciences, Faculty of Agriculture, Food and Environment, The Hebrew University, Rehovot 76100, Israel; z.roth@mail.huji.ac.il (Z.R.); yaron.kressel@mail.huji.ac.il (Y.Z.K.); dorit.kalo@mail.huji.ac.il (D.K.); 2 Israel Cattle Breeders Association, Caesarea 38900, Israel; yaniv@icba.co.il

**Keywords:** fertility, heat stress, disease, GnRH, estrus onset

## Abstract

**Simple Summary:**

We used an automatic activity-monitoring system to determine onset of estrus in dairy cows. Within 5 h of onset, we administered a single injection of GnRH analogue to improve fertility during the summer and autumn. The treatment increased pregnancy per insemination during the autumn, but not in the summer. The subgroups for which the treatment specifically tended to improve conception risk during the autumn were: mature (2nd plus parity) cows and cows with uterine disease and ketosis after calving. Detection of estrus onset by activity monitoring and GnRH administration shortly thereafter could be incorporated into a synchronization program, to improve fertility of positively-responding subpopulations of cows.

**Abstract:**

We examined gonadotropin-releasing hormone (GnRH) administration at onset of estrus (OE), determined by automatic activity monitoring (AAM), to improve fertility of dairy cows during the summer and autumn. The study was performed on two dairy farms in Israel. The OE was determined by AAM recorded every 2 h, and a single im dose of GnRH analogue was administered shortly after OE. Pregnancy was determined by transrectal palpation, 40 to 45 d after artificial insemination (AI). Conception risk was analyzed by the GLIMMIX procedure of SAS. Brief visual observation of behavioral estrus indicated that about three-quarters of the events (*n* = 40) of visually detected OE occurred within 6 h of AAM-detected OE. Accordingly, the GnRH analogue was administered within 5 h of AAM-detected OE, to overlap with the expected endogenous preovulatory LH surge. Overall, pregnancy per AI (P/AI) was monitored over the entire experimental period (summer and autumn) in 233 first, second or third AI (116 and 117 AI for treated and control groups, respectively). Least square means of P/AI for treated (45.8%) and control (39.4%) groups did not differ, but group-by-season interaction tended to differ (*p* = 0.07), indicating no effect of treatment in the summer and a marked effect of GnRH treatment (*n* = 58 AI) compared to controls (*n* = 59 AI) on P/AI in the autumn (56.6% vs. 28.5%, *p* < 0.03). During the autumn, GnRH-treated mature cows (second or more lactations), and postpartum cows exhibiting metabolic and uterine diseases, tended to have much larger P/AI than their control counterparts (*p* = 0.07–0.08). No effect of treatment was recorded in the autumn in first parity cows or in uninfected, healthy cows. In conclusion, administration of GnRH within 5 h of AAM-determined OE improved conception risk in cows during the autumn, particularly in those exhibiting uterine or metabolic diseases postpartum and in mature cows. Incorporation of the proposed GnRH treatment shortly after AAM-detected OE into a synchronization program is suggested, to improve fertility of positively responding subpopulations of cows.

## 1. Introduction

Among the stresses that disrupt dairy cow fertility, summer heat stress is a major cause of low conception rate in lactating cows. Average pregnancy per AI (artificial insemination; P/AI) in dairy farms in Israel from 2015 to 2020 dropped among mature cows from winter to summer and autumn (37% to 21% and 27%, respectively, Israel Herd Book). Similar trends have been recorded worldwide [1]. Among the aspects associated with impairment of reproduction by thermal stress, alteration of gonadotropin and steroid levels and disruption of preovulatory follicle and corpus luteum (CL) functions have been documented (for reviews see [2,3,4]).

Abnormal preovulatory luteinizing hormone (LH) surge may lead to disruption of normal ovulation and other functions. In women, low preovulatory LH surge has been associated with disruption of oocyte maturation [5]. Follicle tissues obtained from heat-stressed cows secreted lower levels of steroids [6], and environmental heat stress has been associated with reduced follicular estradiol secretion [7,8,9]. Thus, impairment of the events leading to ovulation may cause fertilization failure. Other studies have shown that in cows under summer heat stress, the gonadotropin-releasing hormone (GnRH)-induced LH surge is lower than in cooled cows [10]. Consequently, heat stress lowers ovulation and fertilization rates [11,12,13]. Similarly, disease-related stress impairs the hormonal cascade of events leading to ovulation [14]. Stress caused by production diseases in high milk-producing cows increases problems of low fertility. Milk yield was associated with an increased incidence of uterine infection; the latter is associated with slow growth of the dominant follicle and low production of estradiol, known to contribute to reduced fertility [14]. Subclinical mastitis is recorded in about 20–40% of cows postpartum [15]. We recently found that one-third of subclinical mastitic cows exhibit delayed ovulation, low follicular estradiol and delayed preovulatory LH surge, leading to lower P/AI in subclinical mastitic cows relative to uninfected controls [16,17,18].

Preovulatory LH surge peaks within a few hours of the onset of estrus (OE; [19]). A single im dose of GnRH analogue at estrus increases peak LH surge and is subsequently associated with increased progesterone level following ovulation [20,21]. In light of these findings, a single dose of GnRH during estrus, to synchronize the preovulatory LH surge and ovulation with timing of AI, has been suggested. A study from Mississippi on a limited number of cows [22] reported that administration of GnRH analogue during estrus improves summer conception rate relative to untreated controls (28% vs. 17%). In another study [21], a GnRH analogue was given within 3 h of OE to synchronized cows with two doses of PGF_2α_ administered 14 d apart. Results showed a marked increase in P/AI during the summer and autumn (July–October) in treated cows relative to untreated controls (51.7% vs. 35.1%), whereas no effect was noted in the winter. Given these positive findings, it was reasonable to conclude that GnRH administration shortly after OE can improve summer and autumn fertility. However, it was also clear that an approach based on day-long visual estrus detection was not feasible for commercial farms.

The use of automatic activity-monitoring (AAM) systems is becoming more common on commercial farms worldwide. The AAM for estrus relies on frequent recordings, thereby eliminating the need for visual estrus detection. This approach has been found to be highly efficient for reproductive management based on AI following estrus [23]. Due to this technological advance, GnRH administration shortly after AAM-detected OE has become feasible. The first objective of the current study was to compare the timing of visually detected OE to that of AAM-detected OE, so as to determine the precise timing of GnRH administration that will likely coincide with the natural preovulatory LH surge. The second objective was to examine whether a single dose of GnRH analogue given shortly after AAM-detected OE improves fertility of lactating cows during the summer and autumn.

## 2. Materials and Methods

### 2.1. Animals

The experiment was conducted during the summer (July–September) and autumn (October–November) on two commercial dairy farms located in the coastal plain in Israel. The study was approved by the local Ethics Committee of the Hebrew University (code number: IL 623/15). Meteorological data were collected from nearby stations for both herds. Summer and autumn mean daily maximal and minimal air temperature, relative humidity and temperature humidity index (THI)—calculated according to the equation: THI = (1.8 × AT + 32) – ((0.55 − 0.0055 × RH) × (1.8 × AT − 26)) where AT = ambient temperature, RH = relative humidity [24]—are presented in Table 1.

Cows received a total mixed ration containing 1.77 Mcal/d, 16.5% protein and 32% NDF, ad libitum. A total of 208 cows were included in the entire dataset; more details on distribution of AI and cows are given in the Results section. Cows were milked three times daily, yielding an average 12,600 kg milk over 305 d. Cows were routinely checked by the herd veterinarian and body condition score (BCS) was recorded after calving (overall mean of 3.2) and at peak lactation [25]. Cows were held in open shaded structures in both herds. Postpartum uterine disease, mastitis and metabolic diseases, mainly ketosis, were diagnosed and recorded, as previously detailed [17,26]. Cows were treated by the veterinarian according to routine protocols. Cows underwent first AI following detection of estrus according to herd policy, i.e., between 70 and 90 d in milk according to parity and BCS. Pregnancy was diagnosed by transrectal palpation 40 to 45 d post-AI. Cows were cooled during the summer by a sprinkling and ventilation cooling system, as previously described [27,28]. Cooling was applied in the waiting area before milking, and along the feeding lines. Cows were cooled for about 6 h during a 24-h cycle.

### 2.2. Experimental Facilities

In both herds, cows were equipped with an AAM system (Heatime, SCR Dairy, Netanya, Israel). The device is placed on the cow’s neck to monitor neck movements. Data are frequently recorded (every 20 min), and are summed and stored every 2 h. An increase in monitored activity above a specific threshold for an individual cow sends out an estrus alert; the point in time at which the cow was first recorded as being in estrus was defined as OE. A professional technician routinely performed AI once a day in the morning hours. A cow recorded in estrus during the morning hours was inseminated on the same day; if OE was recorded in the afternoon or evening, the cow was inseminated the next morning.

### 2.3. Behavioral Study

A short visual observation of behavior was performed to compare individual timing of OE determined by AAM to that determined by visual detection (*n* = 40 cows). Visual observation was performed to record the exact timing of OE, with standing to be mounted as the main criterion. In addition, cows exhibiting several mountings of other cows or head mountings of other cows were also identified as being at OE [29]. For each cow, visual OE data were compared to the respective AAM-detected OE data.

### 2.4. Fertility Study

Cows in their first to third AI were assigned to the control or treatment group on alternate days of the week, according to the day on which they exhibited estrus. GnRH was administered during working hours (04.00–22.00 h). This management simplified the experimental procedure for the herd crew. Cows in the treatment group that were identified by the AAM system as being at OE were treated within 5 h with 2 mL of im-administered GnRH analogue (200 μg Gonadorelin, Gonabreed, Parnell Laboratories, Alexandria, Australia).

### 2.5. Statistics

The data from the behavioral experiment are presented in the form of descriptive statistics. For each cow, visually detected OE and AAM-detected OE were compared. The difference, in hours, between the two observations was recorded. The AAM-detected OE was defined as time zero.

Data from the fertility experiment were analyzed by a multivariable model that was designed with a logistic model statement using the GLIMMIX procedure of SAS (version 9.2, SAS Institute Inc., Cary, NC, USA); insemination outcome was the dependent variable, as previously described [17,26]. Pregnancy per first, second and third service and P/AI for all services were calculated as number of pregnancies divided by number of AI.

An initial model included, among others, the variables herd and AI number. However, an initial analysis showed that these variables and their interactions with treatment do not differ between groups, and they were therefore excluded from further analyses. Two models were applied. The first consisted of the entire dataset in summer and autumn (*n* = 233 AI), and was analyzed with the general form:

P/AI = intercept + treat + parity + season + metabolic diseases + uterine diseases + mastitis + treat × parity + treat × season + treat × metabolic diseases + treat × uterine diseases + treat × mastitis + error

where: P/AI = lnP/(1−P), P = probability of pregnancy; treat = AI following GnRH treatment or untreated control; parity = first, or second and more lactations; season = AI in the summer (July–September) or autumn (October–November); metabolic diseases = metabolic health status postpartum; uterine diseases = uterine health status postpartum and mastitis = uninfected or subclinical groups.

The second model consisted of the autumn data only (*n* = 117 AI). It was analyzed with the general form:

P/AI = intercept + treat + parity + metabolic diseases + uterine diseases + mastitis + treat × parity + treat × metabolic diseases + treat × uterine diseases + treat × mastitis + error (see description of variables for first model).

For detailed definitions of the diseases, see Lavon et al. [17,26]. Only interactions of all variables with group treatments were included. All variables were considered fixed effects. Probability of conception for the level of a specific variable included in the model was based on least square mean (LSM) values. To compare levels within a variable, we ran the Bonferroni adjustment for multiple comparisons. Values of *p* < 0.05 were considered significant, and values of *p* < 0.10 as tending toward significance.

## 3. Results

### 3.1. Behavioral Study

A comparison of the timing of AAM-detected OE (defined as time zero) and visually detected OE is presented in Table 2. In about half of the cows, visually detected OE coincided (±2 h) with AAM-detected OE, and an additional ca. one-quarter of the cows presented visually detectable OE within 3–6 h of the AAM-detected OE (Table 2). Given that about three-quarters of the cows thus presented standing estrus within 6 h of the OE determined by AAM, treated cows were administered GnRH within 5 h of AAM-detected OE.

### 3.2. Fertility Study

#### 3.2.1. Treatment Effect during the Entire Experimental Period (Summer and Autumn)

A total of 116 and 117 AI were recorded for the treatment and control groups, respectively, consisting of 57 and 59, 33 and 37, and 26 and 21 first, second, and third AI, respectively. Mean days from calving to first, second and third AI were quite similar for treatment and control groups: 88 and 93, 118 and 130, and 158 and 153, respectively. A small number of cows (*n* = 11 and 16 for treatment and control groups, respectively) that had been subjected to AI, had not conceived and expressed estrus were randomly assigned to the experimental groups, and analyzed as different experimental units. Collectively, a total of 233 AI performed on 208 cows were included in the entire dataset.

The analysis of the entire experimental period (summer and autumn) is presented in Table 3. Main effect of treatment on P/AI did not differ from the control (45.6 vs. 38.1%, respectively; Table 3). Summer and autumn’s main effects on P/AI did not differ. Moreover, none of the other main effects: lactation (first vs. second and more), BCS (high vs. low) and the three diseases (healthy vs. sick) differed (data not presented). A tendency toward a group-by-season interaction noted in the GLIMMIX model (*p* = 0.07) indicated no effect of treatment vs. control in the summer (P/AI of 40 vs. 45.6%) and a large, 20% unit difference in P/AI between GnRH-treated and control groups in the autumn (51.3 vs. 31.2%). No interactions of variables parity, BCS or diseases (ketosis, metritis and mastitis) with group were detected (Table 3).

#### 3.2.2. Treatment Effect during the Autumn

**Figure 1:**
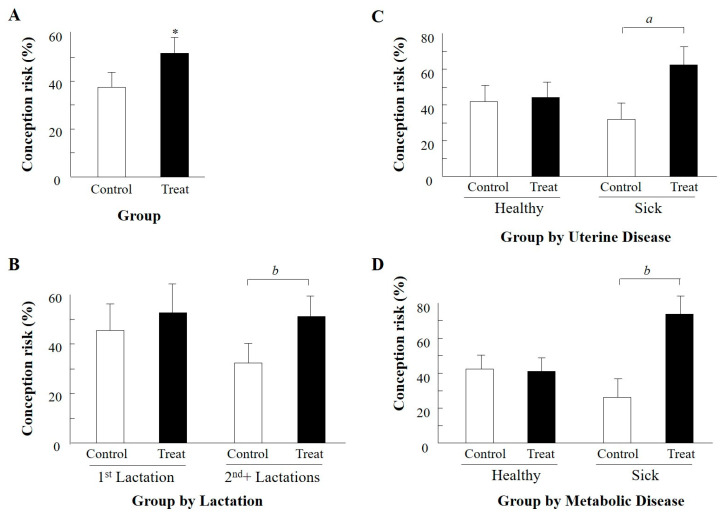
Effect of GnRH analogue administered within 5 h of onset of estrus (OE) on mean actual conception risks of control relative to treatment groups during the autumn only. (**A**) = main effect group, treatment vs. control; (**B**) = group by parity interaction, 1^st^ vs. 2+ lactations; (**C**) = group by uterine disease interaction, healthy vs. sick groups; (**D**) = group by metabolic disease interaction, healthy vs. sick groups. Interactions with a tendency toward significance are presented. Numbers of AI are presented in Table 4. Data are mean ± SEM. Treat = treatment. * *p* < 0.03; ^*a*^ *p* = 0.07; ^*b*^ *p* = 0.08.

Because the group-by-season interaction indicated a large difference between groups in the autumn and no difference in the summer, we further analyzed the data for the summer and autumn separately. Because no significant effects were detected during the summer for any variable or its interaction with group, the summer data are not presented. Accordingly, we concentrated on the effects of treatment during the autumn and results are presented in Table 4 and Figure 1. During the autumn, a total of 117 AI, 58 and 59 AI in treated and control groups, respectively, were performed on 105 cows. Of these, 12 cows that were AI and returned to estrus were randomly assigned to the experimental groups, and analyzed as different experimental units, as described above. The LSM values are presented in Table 4, and actual means of selected significant or tending toward significant variables and interactions are presented in Figure 1. We found a significant main effect of group (*p* < 0.03), reflecting higher P/AI for the treated cows compared to their untreated control counterparts (Table 4 and Figure 1A). Group-by-parity interaction (*p* = 0.08) indicated a much larger tendency for LSM of conception risk in the treated vs. control cows in mature (second and more lactations), but not in first-lactation cows (Table 4, Figure 1B). Group-by-uterine disease interaction (*p* = 0.07) and group-by-metabolic disease interaction (*p* = 0.08) showed a tendency toward a large difference between LSM of sick, treated subgroups and their sick, control counterparts (63.6 vs. 22.7% and 70.3 vs. 23.0%, respectively, Table 4). The respective actual means are presented in Figure 1C,D. No differences were detected between treated and untreated healthy, uninfected subgroups (Table 4, Figure 1C,D).

## 4. Discussion

The present study shows that a single dose of im-administered GnRH analogue at the expected time of preovulatory LH surge, shortly after OE, can improve conception risk during the autumn, but not in the summer. In the autumn, GnRH-treated mature cows, and those with metabolic and uterine disease postpartum exhibited a tendency toward much higher P/AI than their untreated counterparts. In contrast, first lactation cows and healthy cows did not respond to the GnRH treatment. Cows under disease-induced stress might have impaired hormonal secretions, which in turn attenuate the process of preovulatory LH secretion and ovulation [14]. Thus, it is possible that GnRH administration shortly after OE improves the timing and amplitude of the LH surge, thereby leading to ovulation, resumption of meiosis of the ovulated oocyte and increased fertilization rate. In addition, GnRH-induced normal LH surge may improve normal CL formation and progesterone secretion [30], which is important in supporting the formed embryo.

The current findings contrast with a previous study [21] in which GnRH given shortly after OE increased P/AI in the summer. Differences between studies might be related to the intensity of the summer heat stress and the efficiency of cooling, which was likely better in the previous study than in the current one. It is therefore possible that the effect of treatment is limited when cows are under severe thermal stress. In support of this assumption, GnRH administration had a beneficial effect during the autumn, when cows are no longer exposed to high temperatures; however, a carryover effect of summer heat stress into the subsequent autumn has been reported [31]. During the autumn, lactating cows are still expressing impairments in endocrine function, for instance, low estradiol content in follicular fluid aspirated from preovulatory follicles. In turn, reduced estradiol secretion might lead to low LH secretion, impaired preovulatory LH surge, and disruption of the cascade of events that lead to ovulation. In this case, GnRH administration coinciding with the endogenous LH surge might be beneficial. Another reason for the discrepancy between studies might be differences in the method of estrus detection: visual observation in the previous study [21] vs. AAM in the current one. In this respect, it is well documented that estrus intensity and duration are attenuated under heat stress [32]. Thus, the timing of AAM-detected OE during the summer might differ from that in the autumn. Further investigation is required to confirm this and if validated, a different timing of GnRH injection during the summer may lead to a better response.

Administration of GnRH shortly after OE tended to improve conception risk in cows exhibiting postpartum diseases. This included uterine disease and metabolic disease in the autumn. Postpartum diseases have been linked to reproductive disorders such as delayed resumption of cyclicity, longer period to first AI and more days open, all of which have been suggested to be associated with, among other things, attenuated gonadotropin secretion [33,34,35]. We suggest that GnRH treatment administered shortly after OE can effectively improve disease-impaired functions, promote normal LH surge and ovulation, permit normal fertilization and consequently, improve conception rate. However, further detailed research is required to validate this. Another interesting finding that warrants attention is that GnRH treatment among cows under disease-related stress, e.g., metabolic or uterine diseases postpartum, results in a much higher conception risk than that of their healthy GnRH-treated counterparts. The reason for this is currently unclear. One could speculate that the GnRH treatment interacts with disease-secreted or immune-related metabolites that potentially enhance the positive effect of GnRH on conception.

GnRH treatment shortly after OE was highly effective among mature cows, but no effect was noted in cows in their first lactation. These findings differ from those recorded in our previous study [21], in which GnRH injection given within 3 h of OE detected by visual observation improved conception rate of cows in first lactation. The reason for this discrepancy is unknown, but it might be related to differences in experimental design. In a previous study [21], cows were synchronized prior to GnRH injection, whereas the cows in the current study were not. It is possible that cows in first lactation respond better to the synchronization protocol.

The current study was based on a relatively small number of AI. Therefore, the data should be interpreted with caution. Nevertheless, the novel approach of using AAM to determine OE and the significant improvement of fertility by the GnRH treatment as main effect during the autumn are sound. Similarly, the tendency toward significance (i.e., possibly significant with a larger dataset) of treatment interactions with diseases and parity reflects large and real differences between GnRH-treated cows and untreated controls. Additional research is required to further validate and strengthen our findings and extend the study to other seasons, particularly winter.

The use of AAM systems for estrus detection is on the rise, making automatic detection of OE feasible for the dairy industry. The current GnRH treatment comprised two components: (i) determination of OE by AAM, and (ii) administration of the GnRH dose to the identified cow. The use of AAM to determine OE seemed to be satisfactory, as we found about three-quarters of the cows in standing estrus from 2 h before to 6 h after AAM-detected OE. Aungier et al. [19] found that visually and AAM-detected OE coincide in time of appearance. They, and others, also found that the LH surge occurs within only a few hours after AAM-detected OE [36,37]. Although beyond the scope of this study, based on the above, it seems that in most of the cows in our study, the GnRH dose likely coincided with the endogenous LH surge. For the second component of the treatment, injection of the GnRH dose as part of an estrus-synchronization protocol seems to be a good option. In fact, in our previous study [21], cows were synchronized by two doses of PGF_2α_ given 14 d apart. Estrus synchronization has two advantages: (i) manifestation of estrus simultaneously in several cows, which stimulates and enhances signs of estrus [38]; and (ii) more efficient and easier management in terms of tracking the cows at OE and injecting the GnRH dose. One attractive synchronization program option is the Select Synch protocol [39]: GnRH administered on day 0, PGF_2α_ administered 7 d later, and the GnRH treatment given within 5 h of AAM-detected OE. Taken together, synchronization followed by AAM detection of OE should enable efficient defining of the accurate timing for GnRH administration.

## 5. Conclusions

The online AAM system can automatically detect OE in dairy cows, which may serve as a marker for GnRH administration coinciding with the endogenous LH surge. This, in turn, may promote a normal LH surge that consequently, will increase conception risk in cows under stress. We found that GnRH-treated cows during the warm/hot season, particularly in the autumn, exhibit increased conception risk. Regarding disease-related stress, GnRH-treated cows with uterine or metabolic disease postpartum showed improved conception risk relative to their untreated counterparts, whereas healthy cows did not respond to the GnRH treatment. In addition, mature cows treated with GnRH showed increased conception risk, whereas cows in first lactation did not. Detection of OE by the AAM system and GnRH administration shortly thereafter could be incorporated into a synchronization program, and be applied to positively responding subgroups of cows. More studies are required to further validate this approach during warm and hot seasons, and to examine the proposed treatment with cows under disease stress, particularly in the winter season.

## Figures and Tables

**Table 1:** Mean climatic conditions during the summer (July–September) and autumn (October–November) for the farms located in the coastal plain in Israel.

	Daily Maximal	Daily Minimal
Summer		
Ta (°C)	30.5	22.4
RH (%)	83.2	56.5
THI	79.9	70.9
Autumn		
Ta (°C)	25.1	16.3
RH (%)	82.8	52.3
THI	72.0	60.9

Ta = air temperature; RH = relative humidity; THI = temperature humidity index.

**Table 2:** Distribution of lactating cows based on time interval between visual detection and automatic activity monitoring (AAM) detection of onset of estrus (OE) ^1^.

Interval between Visually Detected OE and AAM-Detected OE (t = 0)	*n*	%
−8 to −3 h	6	15
−2 to +2 h	19	48
+3 to +6 h	10	24
+7 to +12 h	5	13

^1^*n* = 40 cows; 5 cows visually detected in standing estrus were not recorded by the AAM device.

**Table 3:** Generalized mixed model (GLIMMIX) used to estimate the effect of GnRH administration at onset of estrus on probability of conception over the entire experimental period (summer and autumn). Group and season, and interactions with group are presented.

Variable ^1^	Level	*n*	LSM ^2^	SE	^3^*p*-Value
Group	Control	117	38.1	6.2	-
	Treat	116	45.6	5.9	0.4
Season	Summer	116	42.9	5.5	-
	Autumn	117	40.9	5.6	0.79
Group by Season ^4^	Control × Summer	58	45.6	8.1	-
	Treat × Summer	58	40	7.4	1
	Control × Autumn	59	31.2	7.2	-
	Treat × Autumn	58	51.3	7.5	0.44
Group by Lactation	Control × Lac 1	43	46.4	10	-
	Treat × Lac 1	40	43.6	9	1
	Control × Lac 2+	74	30.6	5.6	-
	Treat × Lac 2+	76	47.6	6.3	0.33
Group by BCS	Control × High	57	39.8	7.7	-
	Treat × High	70	42.6	6.4	1
	Control × Low	60	36.4	8.1	-
	Treat × Low	46	48.6	9	1
Group by Metabolic diseases	Control × No	76	42	7	-
	Treat × No	81	40.1	6.6	1
	Control × Yes	41	34.4	8.5	-
	Treat × Yes	35	51.2	9.3	1
Group by Uterine diseases	Control × No	70	44.1	7.7	-
	Treat × No	77	45.7	7.6	1
	Control × Yes	47	35.2	7.8	-
	Treat × Yes	39	45.5	8.3	1
Group by Mastitis	Control × No	88	48.8	6	-
	Treat × No	76	49.6	6.5	1
	Control × Yes	29	28.4	8.7	-
	Treat × Yes	40	41.7	8.8	1

^1^ Data consist of 233 AI from two herds. Treated cows received GnRH analogue within 5 h after onset of estrus. Treat = GnRH treatment; Lac = lactation; *n* = number of AI; BCS = body condition score. ^2^ LSM probabilities of conception obtained from GLIMMIX. ^3^ Adjusted by Bonferroni correction for multiple comparisons. ^4^ Group-by-season interaction (GLIMMIX model): *p* = 0.07.

**Table 4:** Generalized mixed model (GLIMMIX) used to estimate the effect of administering GnRH at onset of estrus on probability of conception during the autumn only. Group and interactions with group are presented.

Variable ^1^	Level	*n*	LSM ^2^	SE	^3^*p*-Value
Group	Control	59	26.5	8.3	-
	Treat	58	57.8	8.8	<0.03
Group by Lactation	Control × Lac 1	22	29.5	11.8	-
	Treat × Lac 1	19	56.5	13.4	1
	Control × Lac 2+	37	23.7	7.6	-
	Treat × Lac 2+	39	59.1	9.5	0.08
Group by BCS	Control × High	18	24.9	9.8	-
	Treat × High	29	64	10.8	0.18
	Control × Low	41	28.2	9.5	-
	Treat × Low	29	51.3	11.6	1
Group by Metabolic diseases	Control × No	40	31.8	9.4	-
	Treat × No	39	42.8	9.4	1
	Control × Yes	19	21.8	9.5	-
	Treat × Yes	19	71.4	12.3	0.08
Group by Uterine diseases	Control × No	31	31.9	10.9	-
	Treat × No	34	49.9	10.8	1
	Control × Yes	28	21.7	8.3	-
	Treat × Yes	24	65.5	11.7	0.07
Group by Mastitis	Control × No	48	34.8	13.7	-
	Treat × No	41	59.6	9.4	0.67
	Control × Yes	11	17.3	9.1	-
	Treat × Yes	17	55.9	8.5	0.46

^1^ Data consist of 117 AI from two herds. Treated cows received GnRH analogue within 5 h after onset of estrus. Treat = GnRH treatment; Lac = lactation; *n* = number of AI; BCS = body condition score. ^2^ LSM probabilities of conception obtained from GLIMMIX. ^3^ Adjusted by Bonferroni correction for multiple comparisons.

## Data Availability

The data presented in this study are available on request from the corresponding author. The data are not publicly available for safeguarding individual rights of farm workers and veterinarians.
